# Polyhydroxybutyrate-co-hydroxyvalerate (PHBV) with Phenolic Acids for Active Food Packaging

**DOI:** 10.3390/polym15214222

**Published:** 2023-10-25

**Authors:** Eva Moll, Amparo Chiralt

**Affiliations:** Instituto Universitario de Ingeniería de Alimentos (FoodUPV), Universitat Politècnica de València, Camí de Vera s/n, 46022 València, Spain; dchiralt@tal.upv.es

**Keywords:** ferulic acid, p-coumaric acid, phenolic acids, PHBV, overall migration, specific migration, crystallinity, active food packaging

## Abstract

PHBV films incorporating 3, 6 and 9% ferulic acid (FA) or p-coumaric acid (PCA) were obtained by melt blending and compression moulding. The films’ microstructures and thermal behaviours were analysed as well as their mechanical, optical and barrier properties. The overall and specific migration of the materials in different food simulants was also characterised. FA was homogeneously mixed with the polymer, whereas PCA was mainly dispersed as fine particles in the PHBV matrices due to its higher melting point. These structural features promoted differences in the physical properties of the films depending on the compound concentration. As the concentration of both compounds rose, the barrier capacity of the films to oxygen, and to a lesser extent water vapour, was enhanced. While FA promoted the extensibility of the films, 9% PCA enhanced their brittleness. Both compounds affected the crystallisation pattern of the polymer, promoting smaller crystalline formations and a slight decrease in crystallinity. Although the overall migration of every film formulation was lower than the overall migration limit (OML), the release of active compounds was dependent on the food simulant; almost total release was noted in ethanol containing simulants but was more limited in aqueous systems. Therefore, these films could be used as food contact materials, contributing to extending the food’s shelf life.

## 1. Introduction

The accumulation of plastic materials in marine and terrestrial ecosystems is currently a major issue, greatly aggrieved by single use plastics [1]. The massive quantity of food packaging used by the food sector makes it one of the main contributors to this large amount of waste [2]. Nevertheless, food packaging is necessary for the preservation of quality and safety, extending shelf lives and reducing food waste. Therefore, the food industry requires the development of sustainable and biodegradable packaging systems which exert no negative impact on ecosystems. In the last 20 years, significant efforts have been made with the intention of substituting petroleum-based persistent polymers for biobased and biodegradable materials for food packaging purposes [3,4]. Of these materials, bio-polyesters produced by microorganisms as energy and carbon reserves [5,6,7], such as polyhydroxyalkanoates (PHAs), are of special interest since these have functional properties close to polyethylene terephthalate (PET), being biodegradable in a natural environment. Additionally, these can be obtained from food waste culture media within the framework of a circular economy.

Of the PHAs, poly(3-hydroxybutirate-co-3hydroxyvalerate) (PHBV) is a thermoplastic polyester used in the formulation of the packaging of several products, with many studies into its usage, performance and biodegradability [5]. Nevertheless, very few studies have addressed the use of bioactive compounds in the polymer blend in order to obtain active packaging materials for food applications [8,9]. Active packaging should have an active, sustained influence on the properties of the packaging in order to maintain food quality and safety and extend food shelf lives [10]. The incorporation of naturally occurring compounds with antioxidant and/or antimicrobial properties into the polymer matrix may contribute to an improvement in the performance of the material while also providing it with a better preservation capacity as food packaging, thus helping to extend the product shelf life and reduce food waste.

Phenolic compounds are secondary metabolites produced by plants with known antioxidant and antimicrobial activities, and with a proven thermal stability above the melting temperature of some biodegradable polyesters [11,12,13]. Incorporating these compounds into the polymer matrix can also contribute to its thermal stability, which makes its reuse or recycling more feasible [14], while their release into the food system with which it is in contact may favour product stability against oxidative or microbial spoilage [15,16,17,18]. Additionally, some phenolic compounds, such as catechin and gallate derivatives, have been proposed as polyester stabilisers or indicators of ageing, having greater thermal and oxidative stabilities in polymer matrices [11,19]. Generally, the incorporation of phenolic compounds into PHA matrices leads to an increase in both the glass transition temperature (T_g_) and the thermo-degradation temperature, as well as crystallization disruption; these have been attributed to a crosslinking effect based on the interchain hydrogen bonds of phenolic hydroxyls and the carbonyl groups of polyesters [20,21,22].

Phenolic acids, such as ferulic and p-coumaric acids, exhibit proven antioxidant and antimicrobial capacities depending on their molecular structure [13,23,24]. Specifically, antioxidant, antibacterial, anti-inflammatory and other health-promoting properties have been reported for ferulic and p-coumaric acids isolated from different plant products [25,26,27,28,29,30,31]. Several studies have incorporated phenolic acids into PHBV matrices, analysing their effect on the properties and the biodegradability of the polymer–phenol blends [8,9,15,32,33]. Nevertheless, no previous studies have been found on the incorporation of ferulic or p-coumaric acid into PHBV matrices and its effect on the material properties for food packaging uses. These phenolic acids are present in a wide variety of plants and are major compounds in some lignocellulosic residues such as rice straw. These acids could be used in the design of active PHBV films for food preservation purposes due to their antioxidant and antimicrobial activities [31,34].

This study aims to obtain new insights into the impact of ferulic and p-coumaric acids on the functional and structural properties of PHBV, its thermal behaviour and crystallization, as well as its migration capacity in food systems. To this end, different concentrations (3, 6 and 9%) of each compound were melt blended with the polymer, obtaining films by compression moulding and characterising their relevant properties (barrier, mechanical and optical) as food packaging materials.

## 2. Materials and Methods

### 2.1. Materials

Poly(3-hydroxybutyrate)-3-co-(hydroxyvalerate) or PHBV (Enmat Y1000P) with a hydroxyvalerate fraction of 2% mol. was purchased in pellet form by TianAn Biologic Materials (Ningbo, China). Ferulic acid (FA) and P-coumaric acid (PCA) were provided by Sigma-Aldrich (Saint Louis, MO, USA). Phosphorus pentoxide (P_2_O_5_) and magnesium nitrate (Mg(NO_3_)_2_) from Panreac Química (Barcelona, Spain) were used for conditioning the samples at a controlled relative humidity.

The food simulants used for migration studies, such as ethanol (99.9% purity), glacial acetic acid and isooctane, as well as UV-grade methanol, were acquired from Sigma-Aldrich Chemie (Steinheim, Germany).

### 2.2. Preparation of Films

Prior to the mixing of film components, PHBV was dried in a vacuum oven at 60 °C for 24 h to eliminate the absorbed water. The films were prepared by melt blending the PHBV with different proportions of phenolic acids, as described in [35]. Specifically, 3, 6 or 9 g of each acid per 100 g blend was used to obtain the different batches. The melt blending of components was carried out in an internal mixer (Haake PolyLab QC, Thermo Fisher Scientific, Dreieich, Germany) at 170 °C and 50 rpm for 5 min. Blend samples were cold ground and stored in a desiccator with P_2_O_5_ (0% relative humidity) until use. Films were obtained by the thermocompression of 3.5 g of each blend using a hydraulic hot plate press (LP20, Labtech engineering, Bangkok, Thailand) by preheating for 5 min at 180 °C, compressing for 4 min at 180 °C and 100 bar pressure and finally cooling for 3 min to about 70 °C. The obtained films were stored at 0% relative humidity until characterisation. PHBV films with different ratios of ferulic acid were named 3FA, 6FA and 9FA, while those with p-coumaric acid were named 3PCA, 6PCA and 9PCA.

### 2.3. Characterisation of Films

#### 2.3.1. Final Concentration of Phenolic Acids in the Films

The final concentrations of both FA and PCA in the films after film processing were analysed by spectrophotometric quantification of the film’s methanolic extracts [36]. Dry film samples (100 mg) were extracted with 10 mL of methanol at 20 °C for 48 h under stirring. Absorbances of the methanolic extracts were measured at 321 and 309 nm for FA and PCA, respectively, using a UV-visible spectrophotometer (Thermo Scientific Evolution 201, Brooklyn, WI, USA), and compared with previously obtained standard curves for each compound. The methanolic extract of the net PHBV films was used as a background. Analyses were carried out in quadruplicate for each sample.

#### 2.3.2. Microstructural Analyses

The microstructure of the different films was analysed by Field Emission Scanning Electron Microscopy (FESEM Ultra 55, Zeiss, Oxford Instruments, Oxford, UK). The samples were stored for 1 week with P_2_O_5_ (0%HR) to ensure dryness and cryofractured by immersion in liquid N_2_ to observe the film cross-section. Then, they were mounted on a support stub and coated with platinum prior to observing them in the microscope at 1.5 kV.

#### 2.3.3. Thermal Analyses

The polymer phase transitions in the different films were analysed by Differential Scanning Calorimetry (DSC823^e^ Star^e^ Mettler-Toledo Inc., Greifensee, Switzerland). The samples (5–10 mg) conditioned to 0% RH in P_2_O_5_ were placed in aluminium crucibles and sealed. The samples were submitted to three steps: heating (10 °C/min), cooling (50 °C) and heating (10 °C) under nitrogen flow between −30 °C and 200 °C. The degree of crystallinity of the polymer was determined from the melting enthalpy, the polymer mass fraction in the blend and the reported value of 100% crystalline PHB (ΔH_0_PHB = 132 J/g polymer) [37].

The thermal stability of the films was analysed by TGA using the TGA 1 STAR^e^ System analyser (Mettler-Toledo, Greifensee, Switzerland). Samples of approximately 5 mg were weighed in alumina crucibles and heated under a N_2_ flow (10 mL/min) at 10 °C/min from 25 to 600 °C.

The analyses were carried out in duplicate for each sample.

#### 2.3.4. X-ray Diffraction (XRD)

The X-ray diffraction spectra of the different films were measured with an X-ray diffractometer (AXS/D8 Advance, Bruker, Karlsruhe, Germany) using Cu-Kα radiation (λ: 1.542 Å, 40 kV, 40 mA), a 2θ scan angle of between 10° and 60° and a step size of 0.04°/min. For each sample, the crystallinity index (CI, expressed as a percentage) was determined from the crystalline peak area divided by the total area under spectra using Origin (OriginLab Corporation, Northampton, MA, USA).

#### 2.3.5. Fourier Transform Infrared Spectroscopy (FTIR)

The FTIR spectra of the films were obtained using a FTIR spectrometer (Agilent Cary 630, Agilent Technologies Inc., Santa Clara, CA, USA) equipped with an attenuated total reflectance (ATR) accessory. The spectra were obtained at a resolution of 2 cm^−1^ in the wavelength range of 4000–500 cm^−1^, performing 32 scans for each spectrum. The analyses were carried out in duplicate on each film.

#### 2.3.6. Tensile Properties

The tensile tests in the different films conditioned at 53% RH were performed following the ASTM D882 standard method [38] using a universal testing machine (Stable Micro Systems TA-XT plus, Haslemere, UK). Briefly, 25 × 100 mm film strips were cut, and their thickness was measured with a digital micrometer (Comecta S.A., Barcelona, Spain) at six random points. The film sample was clamped (5 mm separation) in the tension test clips and stretched at 50 mm/min until break. Henky stress–strain curves were obtained and the elastic modulus (EM), tensile strength (TS) and elongation at break (%E) were determined from the force–distance curves. Twelve replicates per formulation were performed.

#### 2.3.7. Barrier Properties

The oxygen permeability of the different films conditioned at 53% RH was determined following the ASTM D3985-05 standard method [39] using a permeation analyzer (Model 8101e Oxysense, Systech Illinois, IL, USA). The films were cut to a surface area of 50 cm^2^ and their thickness was measured with a hand-held digital micrometer (Comecta S.A., Barcelona, Spain). The oxygen permeability (OP) was calculated from the oxygen transmission rate (OTR), the film thickness and the partial pressure of oxygen in the instrument. Each sample was measured in duplicate.

The gravimetric method, ASTM E96/E95 M [40], was used to obtain the water vapour permeability (WVP) of the films conditioned at 53% RH using Payne permeability cups (Elcometer SPRL, Hermelle/s Argenteau, Belgium). The film samples were cut (35 mm diameter) and their thickness was measured at six random points. The cups were filled with 5 mL of distilled water and covered with the film. To obtain a 100–53% RH gradient, the cups were placed in a desiccator containing an oversaturated Mg(NO_3_)_2_ solution (53% RH) at 25 °C. The cups were weighed every 2 h for 48 h with an analytical balance (±0.00001 g). The WVP was calculated from the slope of weight loss vs. time data once the steady state was reached, as described by Cano et al. in 2014 [41].

#### 2.3.8. Optical Properties

Internal transmittance (Ti) and CIE-L*a*b*colour coordinates were determined for the different films using a CM-5 spectrocolourimeter (Konica Minolta Co., Tokyo, Japan) through reflectance measurements (from 400 to 700 nm) under white and black backgrounds of known reflectance. The internal transmittance (Ti) and the infinitely thick reflectance (R_∞_) spectra were determined applying the Kubelka–Munk theory for multiple scattering [42]. CIE-L*a*b* coordinates were obtained from R_∞_ spectra by considering illuminant D65 and observer 10°. From the CIE-L*a*b* coordinates, chroma, C_ab_* (Equation (1)), and hue, h_ab_* (Equation (2)), were calculated.

(1) $$C_{ab}^{*}=\sqrt{{\left(a\right)}^{2}+{\left(b^{*}\right)}^{2}}\;\;\;$$

(2) $$h_{ab}^{*}=arctan\left[\frac{a^{*}}{b^{*}}\right]\;\;$$

### 2.4. Migration Analyses in Different Food Simulants

The overall migration (OM) of the films was determined according to the EN-1186 series of European Standards [43,44,45], where it is specified that the overall migration limit for new materials intended for contact with foodstuffs should be 10 mg/dm^2^. The simulants used for these tests are also specified (simulant A: distilled water; simulant B: 3% (*w*/*v*) acetic acid; and simulant C: 10% (*v*/*v*) ethanol, to simulate aqueous foods, and ethanol 95% (*v*/*v*) and iso-octane to simulate fatty foods). The test temperature and time were chosen by considering the worst conditions under which the new material can be exposed, i.e., 40 °C for 10 days, emulating storage at room temperature or below for an unspecified period. For this purpose, 1 dm^2^ film samples were immersed in 100 mL of simulant contained in tubes and kept for 10 days at 40 °C. After this time, the films were removed, and the simulant was evaporated till dryness at 105 °C. The OM is the residual mass after evaporation of the simulant, expressed as mg/dm^2^ of film. Each film sample was analysed in triplicate.

The specific migration of the incorporated phenolic acids was also measured. For this purpose, the dried residue was extracted in methanol for 30 h under stirring and the absorbance was measured in a spectrophotometer (Thermo Scientific Evolution 201, USA) at the maximum wavelength for each of the acids (321 nm for ferulic acid and 309 nm for p-coumaric acid). The FA and PCA concentrations of each sample were obtained using a previously obtained calibration curve relating the concentration of the respective acid in methanol solutions to its absorbance in the ultraviolet region [35]. Pure methanol was used as a blank.

### 2.5. Statistical Analysis

The statistical analysis of the data was performed by an analysis of variance (ANOVA) using Statgraphics Centurion XVII-X64 and using Fisher’s Least Significant Difference at the 95% confidence level.

## 3. Results and Discussion

### 3.1. Structural and Molecular Properties of the Films

The effect of the incorporation of phenolic acids on the film microstructure was evaluated by FESEM and FTIR spectra. The final concentration of active compounds in the thermally processed films was considered in this analysis, as it can affect the arrangement of the components in the matrix. The actual amounts of PCA in the films were 2.90 ± 0.02, 6.10 ± 0.02 and 8.73 ± 0.04 g PCA/100 g of film in the 3PCA, 6PCA and 9PCA samples, respectively (98 ± 6% of the incorporated acid), while ferulic acid was retained in lower amounts, namely 2.68 ± 0.02, 4.54 ± 0.07 and 7.62 ± 0.04 g FA/100 g of film for the 3FA, 6FA and 9FA samples, respectively (85 ± 6% of the incorporated acid). This was also observed in a previous study [35], where the partial degradation of FA during processing was attributed to its greater thermosensitivity. Similar losses in FA were observed by other authors for PLA-based thermoprocessed films or PLA-PHBV blend films [32,46]. In fact, FA melts at 173 °C and degrades between 200 and 240 °C [47]. Therefore, throughout its blending with the polymer at 170 °C and subsequent film thermoforming at 180 °C, it can suffer partial degradation in the liquid state due to local overheating and the shear forces applied in the mixer. In contrast, PCA melts between 210 and 216 °C, simultaneously degrading during melting [47]. Therefore, at the temperature of blending and film formation, PCA was more stable in the solid state, making mixing more difficult and preventing degradation [35].

**Figure 1:**
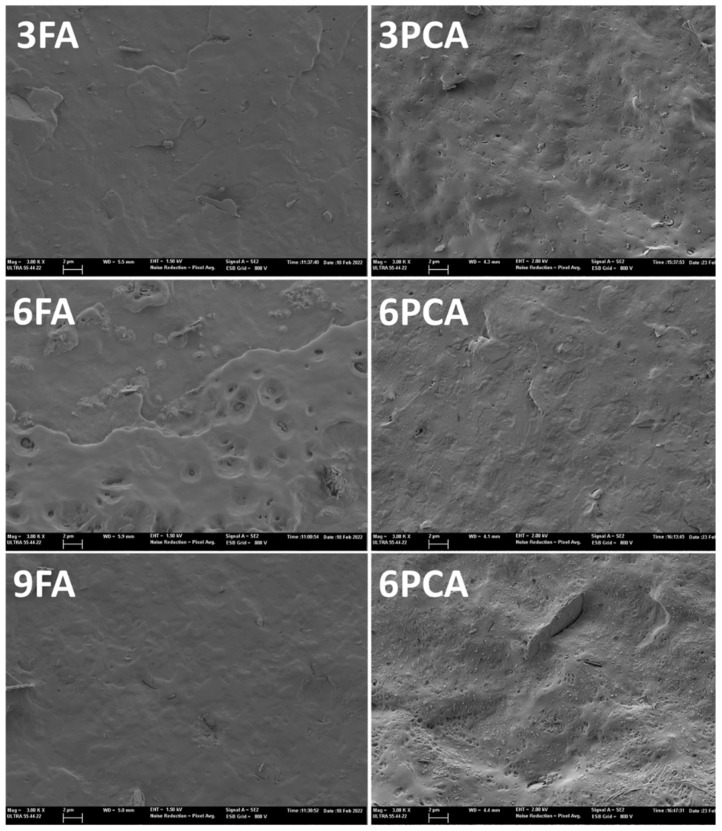
FESEM micrographs of cross-sections of PHBV films with 3, 6 and 9% ferulic acid (3FA, 6FA and 9FA, respectively) or p-coumaric acid (3PCA, 6PCA and 9PCA, respectively). Magnification: 3000×; bar: 2 µm.

Figure 1 shows the FESEM micrographs of the different films, in which the different structural features of each film can be observed. Differences in the melting points of the phenolic acids influenced the microstructure of the film. As PCA remained solid during blending, PCA particles were observed to be dispersed in the polymer matrix, which was more evident at the highest PCA concentration in the films (Figure 1). In contrast, FA in the liquid state was homogeneously mixed with the polymer, and molecular interactions between the phenolic acid and the polymer chain were favoured, e.g., via hydrogen bonds between the phenolic or carboxylic hydroxyls and the carbonyl group of the polyester or -OH end-chain groups [20,21]. Thus, films with FA at any concentration presented a homogeneous cryofracture surface according to the molecular mixture of polymer and phenolic acid. However, in the cross-section of the PCA films, small, scattered particles appeared in the polymer matrix, attributed to PCA particles that did not melt during processing. This was especially noticeable at higher concentrations (6 or 9%) of PCA in the films (Figure 1). Differences in the structural arrangement of phenolic acids in the polymer matrix can alter their functional and thermal properties, as well as the appearance of the films.

**Figure 2:**
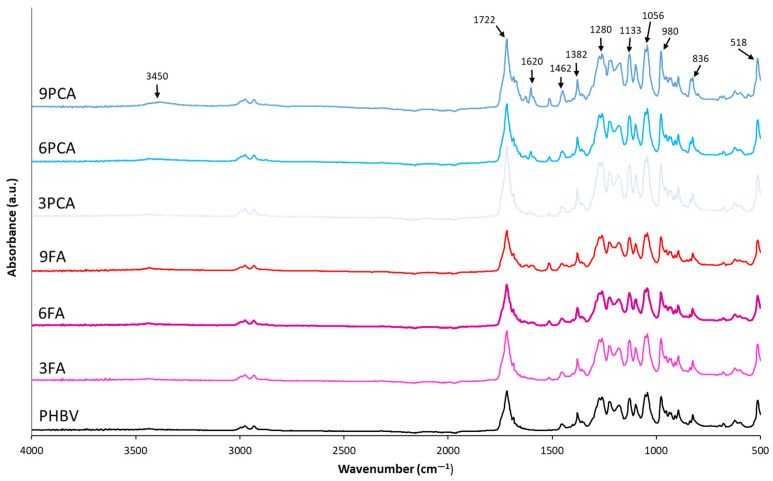
FTIR spectra of the different PHBV films without phenolic acids, 3, 6 and 9% ferulic acid (3FA, 6FA and 9FA, respectively) or p-coumaric acid (3PCA, 6PCA and 9PCA, respectively).

The molecular changes produced in the PHBV matrix due to the incorporation of phenolic acids were evaluated through the FTIR-ATR spectra of the films (Figure 2). These showed the typical spectra of PHBV [48,49,50], as the major component, but small differences could be observed when phenolic acids were incorporated. All the samples showed bands associated with the vibrations of polymer functional groups. Thus, stretching of the C-H group was observed in the region between 836 and 980 cm^−1^, while C-H bending vibrations were reflected in the peaks at 1382 and 1462 cm^−1^ [48,51]. Peaks at 1052, 1133 and 1280 cm^−1^ related to the C-O-C stretching bands also appeared, as well as the characteristic peak of the C=O stretching vibration at 1722 cm^−1^ [50,51,52], which overlaps with the carbonyl vibration of phenolic acids (1670–1691 cm^−1^) when these are present in the films.

Films containing phenolic acids also exhibit new small peaks between 1500 and 1650 cm^−1^, where there are no bands of the major compound (PHBV) in the films. These peaks at 1518, 1584, 1602 and 1630 are similar in films containing both PCA and FA but are much more defined, with a higher relative intensity for films with PCA, especially at the highest concentrations. These can be associated with the C=C bond vibration of the p-substituted aromatic ring and vinyl group [53,54,55,56]. The higher relative intensities of these peaks for films with p-coumaric acid seem to reflect the greater contribution of non-bonded PCA molecules to the polymer, since the compound is mainly present as dispersed particles. In general, films with added phenolic acids show a moderate peak at 3450 cm^−1^, belonging to the absorption band of their hydroxyl group [57,58].

### 3.2. Thermal Behaviour and Crystallinity of the Films

**Figure 3:**
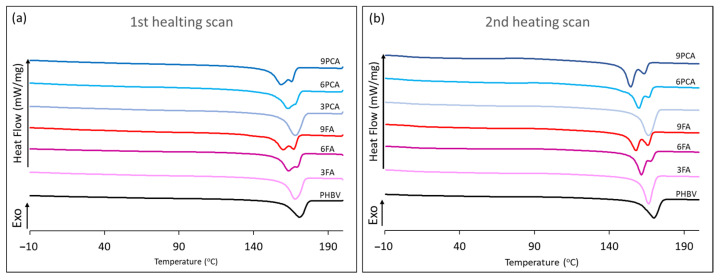
DSC thermograms of PHBV and different formulations with phenolic acids. Effect of concentration and type of acid. First heating step (**a**) and second heating step (**b**).

PHBV exhibits a high degree of crystallinity that results in a narrow processing window, hindering its processability in conventional industrial equipment [59]. Different strategies have been used to overcome this issue, such as blending with other polymers, fillers or compatibilisers [59,60,61]. Blending with phenolic acids could affect the crystallisation of the polymer, inhibiting nucleation and spherulite formation. Previous studies reported an increase in the polymer glass transition, as a result of blending with phenolic compounds, and changes in the crystallisation pattern [8]. Figure 3 shows the thermograms of the different film samples obtained after the first heating, where the behaviour of the thermoprocessed material itself is shown, and after the second heating step of the DSC analysis, in which the material’s thermal history was erased. In both scans, a typical glass transition and melting endotherm of the semi-crystalline polymer were observed, although the presence of phenolic acids affected the thermal behaviour depending on the added compound and its concentration. Table 1 shows the values of the melting temperature (T_m_) (the first peak when two appeared) and enthalpy (∆H_m_) of both heating scans and the glass transition temperature (T_g_) and degree of crystallinity (χ_c_) of the polymer (determined from ΔH_m_ values) of the first heating. FA provoked an increase in the T_g_ values, which was enhanced when its concentration rose in the films, whereas no significant changes were observed with PCA. This varying effect can be attributed to the different compound–polymer interactions established in each case. FA acid could interact more easily with the polymer when it melts during blending, giving rise to polymer chain entanglements through hydrogen bonds, as previously metioned, which would make the molecular mobility more restricted, thus increasing the T_g_ value. These interactions can be promoted by a great number of FA molecules in the blend. In contrast, solid PCA particles in the PHBV matrix did not interact with polymer chains to the same extent and, therefore, no significant changes in the glass transition temperature were observed.

Regarding PHBV crystallisation, at zero and low concentrations of phenolic acids, a single melting peak was observed, whereas from concentrations of 6% upwards of both acids, two overlapped melting peaks appeared, with the second peak being more pronounced as the concentration of phenolics rose. Moreover, the melting temperature of the first peak decreased as the concentration of phenolic acids increased. Therefore, both compounds affected the crystallisation pattern of the polymer depending on their concentration. The appearance of two melting peaks is related to the recrystallisation of the polymer after melting due to the formation of smaller crystalline domains that melt at lower temperatures and the subsequent recrystallisation due to the annealing undergone by the material during heating [62]. In this sense, the first peak could be attributed to the melting temperature of the smaller crystals formed during cooling that melt at lower temperatures. Therefore, as observed in other blends of PHBV [63], phenolic acids promoted the formation of smaller crystalline domains; this effect was more notable when their concentration rose in the blend and when the cooling rate of the material increased [64,65]. This can be attributed to the steric hindrance imposed by the compounds present in the crystallisation of the PHBV chains, reducing the packing efficiency in the crystals [66].

There were also significant differences between net polymer and blends with FA as regards the melting enthalpy per g polymer (Table 1), which suggested that this compound could also limit the crystallisation of the polymer. The differences between the pure polymer and blends were greater in the second heating step, where crystalline domains were formed at a higher cooling rate (50 °C/min), than in the first heating, where the polymer films were cooled more slowly (about 37 °C/min). This suggests that FA delayed the crystallisation rate of PHBV, thus reaching a lower degree of crystallinity when cooled fast. The formation of a hydrogen bond between PHBV and phenols has been described by other authors to reduce the polymer crystallisation [21,22]. PCA has no significant effect on the final crystallisation of PHBV, probably due to the fact that the compound does not mix completely but remains as dispersed particles.

**Figure 4:**
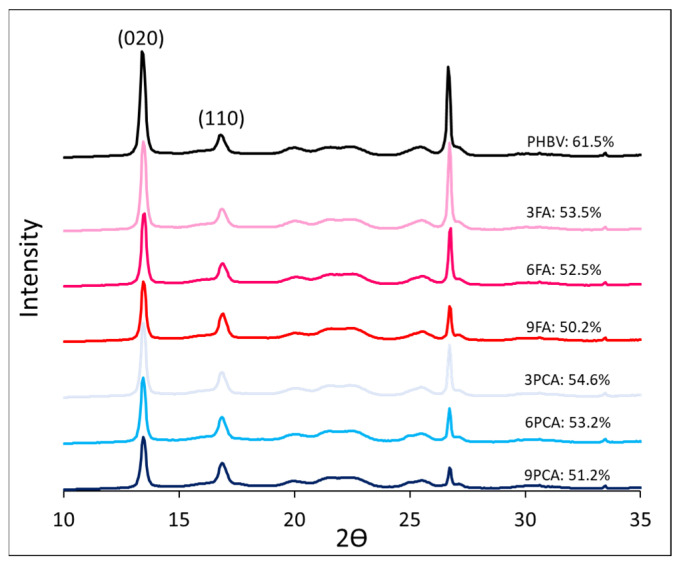
RX diffraction spectra of the films with different formulations, showing the crystallinity index in percentage.

The X-ray diffractograms of the different samples (Figure 4) show the typical crystalline pattern of PHBV [61,67], with two main peaks at 2θ, 13.5° and 17°, which are associated with the (020) and (110) diffraction planes of the α phase and the orthorhombic lattice of PHBV, respectively. The intense peak at 2θ (27°) could correspond to boron nitride, which is incorporated into the commercial polymers as a nucleating agent [61]. No changes in the crystalline pattern were provoked by phenolic acid incorporation at any concentration. Nevertheless, as also deduced from DSC analyses, it promoted a decrease in the degree of crystallinity (χ_c_) that was determined from diffraction intensity data after the deconvolution of peaks (area of crystalline peaks divided by the total area of crystalline peaks and amorphous halo). Thus, phenolic acids affected the kinetics of PHBV crystallisation, hindering crystal formation and reducing the size of crystalline domains and the final crystallinity index in the blends, depending on the cooling rate of the material.

Small differences between the degree of crystallinity obtained from diffractograms and the melting enthalpy may be explained by the dynamic heating in DSC that induces polymer recrystallisation, which affects the total enthalpy values. Nevertheless, in both methods, the phenolic acids could be observed to reduce crystallisation depending on the cooling rate of the material. Although this reduction occurred for both the homogeneously blended FA and the dispersed PCA, it was mainly seen to take place in the former case.

**Figure 5:**
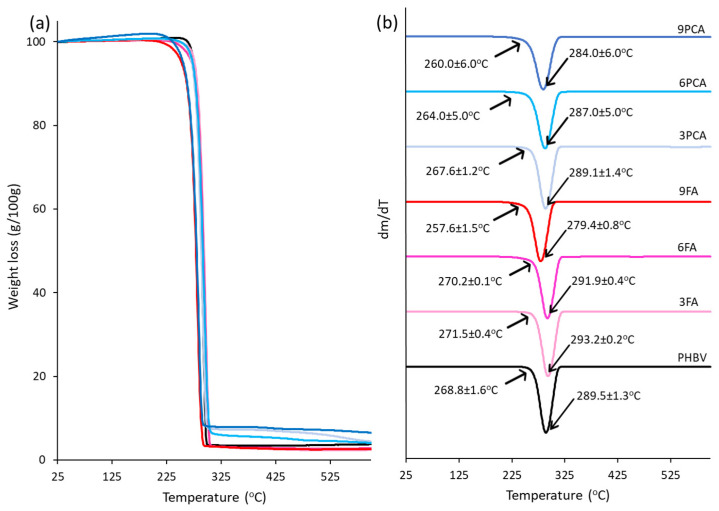
(**a**) TGA and (**b**) DTGA curves of the different films, showing the onset and peak temperatures.

The thermal stability of the polymer was analysed by TGA. Figure 5 shows the mass loss curves as a function of temperature and their derivative curves (DGTA). They are similar to those reported by other authors for PHBV [68,69], with 289 °C as the temperature at which the maximum degradation rate occurs. Although pure FA and PCA degrade at lower temperatures than polymer (210–216 and 200–240 °C, respectively [47]), all films showed a single degradation peak. Nevertheless, these compounds caused some alterations in the onset and main degradation temperatures of the matrix due to the established molecular interactions, changes in the polymer crystallinity or potential hydrolysis effects in the polymer chains that produce less thermostable oligomers. The initial and peak degradation temperatures varied as a function of the compound added and its concentration. For low FA concentrations (3 and 6%), the onset and peak degradation temperatures slightly increased, resulting in a more thermostable matrix, despite the previously mentioned reduction in crystallinity. This could be due to the polymer cross-linking effect in the amorphous phase caused by the hydrogen bonds between ferulic acid and PHBV chains, as observed by other authors when studying films of other polyesters [32,46]. However, for the highest FA concentration (9%), both the initial degradation temperature and the peak degradation temperature decreased, resulting in a less thermally stable material, probably due to the prevailing hydrolysis effect with high acid concentrations. In the case of PCA, both onset and peak temperatures slightly decreased as the compound concentration rose, suggesting no significant cross-linking effect in the amorphous phase; this was coherent with the obtained T_g_ values and was a consequence of the prevalent solid state of the compound dispersed in the matrix. This decrease could be attributed to the small reduction in the degree of crystallinity of the polymer or some hydrolysis effect in the polymer chains.

### 3.3. Mechanical, Barrier and Optical Properties of the Films

The molecular interactions and structural changes caused by the addition of FA and PCA to the matrix resulted in changes in the mechanical, barrier and optical properties, shown in Table 2, together with variations in the mean film thickness. These parameters were also affected by the presence of phenolic acids, which seemed to promote the material flowability, since films tended to be thinner when the concentration of the added compound rose. This behaviour could be attributed to a certain hydrolytic effect of phenolic acids that could reduce the chain length of polymers, promoting the flowability index of the melt during the thermocompression step. However, the reduction in the interchain forces in the presence of the added compound could have a similar effect.

The barrier capacity of the films was also affected by the addition of phenolic acids. Specifically, the water vapour permeability (WVP) slightly decreased when the phenolic acid concentration rose, thus indicating that phenolic acids reduced the water affinity of the polymer, which reduced its water permeation capacity, probably due to the hydrogen bond formation between phenolic hydroxyls and the end-chain hydroxyls of the polyester. This effect was statistically significant (*p* < 0.05) at 9% concentration of both acids, without significant differences between them. Likewise, the addition of phenolic acids led to a significant reduction in the oxygen permeability (OP) in every case, which was more pronounced for p-coumaric acid at 9% (28% reduction). These effects can be attributed to the structural changes caused by the compound in the polymer matrix, which modify both the diffusion of the permeant molecules (water or oxygen) and their chemical affinity with the matrix. In particular, the reduction in OP may be affected by the antioxidant action of phenolic acids, which can act as oxygen scavengers, reducing its passage through the film, as described by other authors for antioxidant organic compounds [70]. This effect is more remarkable in the case of p-coumaric acid, which could be due to its freer form in separate particles, which could better exhibit a oxygen scavenging capacity.

The elastic modulus, the tensile strength and the elongation at break values of the PHBV films were similar to those reported by Requena et al. for this polymer [71]. The incorporation of phenolic acids produced changes in the tensile behaviour depending on the type of compound and its concentration. FA slightly reduced the elastic modulus and resistance to break of the films in line with its concentration, but promoted film extensibility (17% increase at 9%). A similar effect was also observed for PCA at 3 and 6%, but at 9%, the films maintained their initial stiffness and became more brittle with a lower resistance to break. The gain in extensibility of the films could be related to the decrease in their crystallinity or to the partial hydrolysis effect provoked by the acids. Nevertheless, the greater brittleness of the films with 9% PCA could be attributed to the higher ratio of dispersed particles in the film, as shown in FESEM images (Figure 1), acting as a reinforcing filler that promotes stiffness but favours breakage at lower deformations.

**Figure 6:**
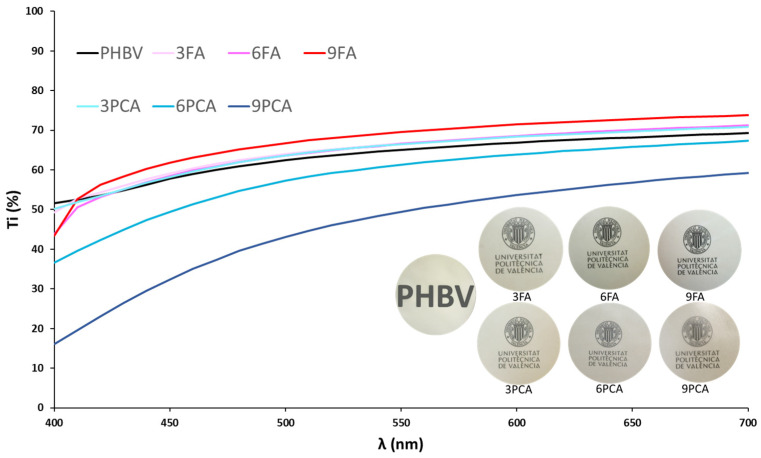
Internal transmittance and photographs of film appearances.

The presence of dispersed PCA particles in the films also caused a decrease in the film transparency, observable to the naked eye and quantified by the internal transmittance (Ti), as shown in Figure 6. As the PCA concentration rose in the films, Ti decreased; this effect was highly remarkable for 9% PCA due to the light scattering effect provoked by dispersed particles. In contrast, the addition of FA did not significantly change the transparency of the films, coherently with its homogenous mixture with the polymer, while it slightly promoted Ti values and film transparency, which may be attributed to the changes induced in the refraction index of the polymer matrix, especially at a concentration of 9%. The changes in light–film interactions promoted by the phenolic acids also affected the colour parameters, shown in Table 2. The slightly more transparent films containing FA became slightly darker, with a more saturated and redder (lower hue) colour as the FA concentration rose, also due to the contribution of the natural colour of the compound. In contrast, for less transparent films (those containing PCA), this effect was mitigated, as they became lighter and less saturated in colour, especially for 9% PCA, which was reflected in the most significant decrease in film transparency.

Therefore, differing concentrations of FA and PCA in the PHBV films provoked modifications in their physical properties which depended greatly on the structural arrangement of the compounds in the polymer matrix. FA was homogeneously mixed with the polymer, whereas PCA was mainly dispersed within the polymer matrix due to the fact that melt blending was hindered by its higher melting point. The dispersion of PCA reduced the compound–polymer chain interactions but produced a reinforcing filler effect depending on the compound concentration. These differences could also affect the migration of the compound from the matrix to the food systems to develop their antioxidant or antimicrobial effects, as discussed in the next section.

### 3.4. Migration in Food Simulants

An important aspect of newly developed packaging materials intended for contact with food is the migration of the film compounds. This is regulated by UNE-EN 1186-1:2002 [43] and UNE-EN 1186-3:2022 [45], which specify both the overall migration limit (OML = 10 mg/dm^2^) and the test conditions (food simulants, temperature and contact time) to determine the overall migration (OM). For active packaging materials, Regulation (EC) No 450/2009 [72] establishes that the amounts of active components released should not be taken into account in the determined OM values, as they are not part of the passive material. Therefore, the fraction of the migrated residue corresponding to the active component (AF or PCA) was quantified spectrophotometrically in the methanolic extract of the residue obtained from each simulant. Nevertheless, their use as active compounds for food contact materials should be authorised by the European Food Safety Authority (EFSA) according to Regulation (EC) No 1935/2004 [73].

**Figure 7:**
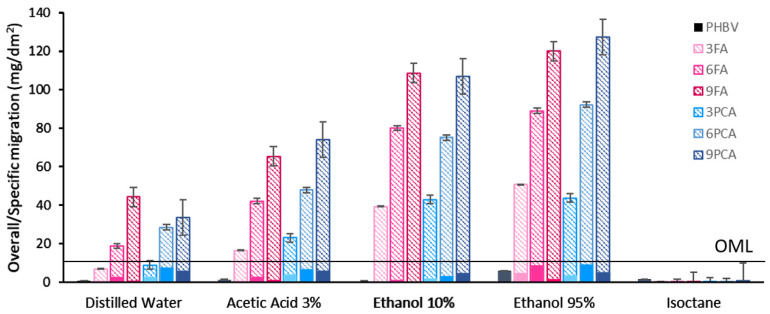
Overall/specific migration (mg/dm^2^) of films in different food simulants of each film: non-solid bars represent the active compound migration and solid bars the total migration which was less the migration of the active compound. The overall migration limit (OML, 10 mg/dm^2^) is shown as a line (-).

All the films were well preserved in every simulant at 40 °C throughout the test duration, and the OM values in different simulants are shown in Figure 7, in which the active compound migration is represented as a non-solid bar and the difference with respect to the total migration values represent the OM values (solid bars) that must be lower than the overall migration limit (OML: 10 mg/dm^2^). In no case was the OML reached, with the OM being less than 10 mg/dm^2^ in the different film formulations for all the solvents used (food simulants). Specifically, the total migration values in isooctane were very limited for every film formulation (less than 1.2 mg/dm^2^), as reported by several studies on PHBV [15,74,75] as well as other polyesters, such as PBSA [76] or PLA [77]. In this case, the specific migration of active compounds was not determined since, additionally, the solubility of phenolic acids in isooctane is very low [78].

**Figure 8:**
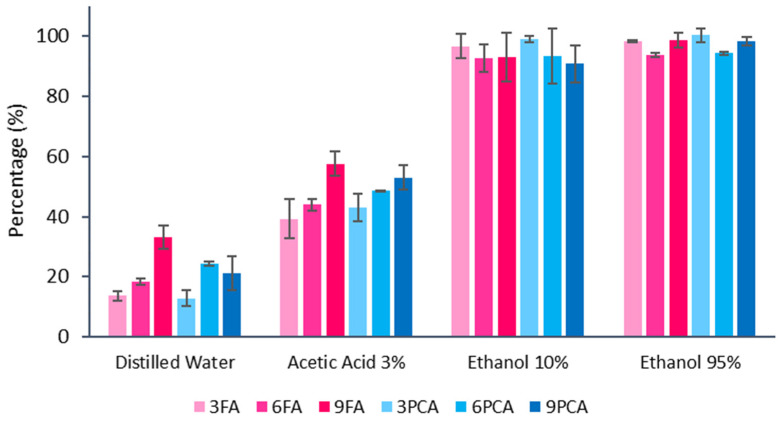
Mean values and standard deviations of the percentage release of the phenolic acids incorporated into each film in the different food simulants.

The percentage release of phenolic acids with respect to the initial content of each film was determined and shown in Figure 8 for the different simulants used. The kind of simulant greatly affected the final release of active compounds. Simulants containing ethanol delivered higher amounts of phenolic compounds from the matrix, with a practically total migration of both active compounds, whereas in distilled water or acidic water, the release was between 12 and 33% or 40 and 60% of the film content, respectively, depending on the compound concentration in the film. The migration of the active compound at equilibrium time (as simulated in the test) is related to the solubility of the phenolic acids in each solvent and their affinity with the polymer matrix (partition coefficient). The ability of each solvent to swell the polymer matrix and modify the overall compound interactions will also affect the active compound migration [35]. The solubility of both acids in ethanol was much greater (about 200 times) than in water [47,79] and thus increased when the ethanol ratio rose in the ethanol–water mixtures [80]. Additionally, ethanol promotes the swelling and degradation of the polyester matrix [81,82], which will affect compound release. The compound concentration in the film has a remarkable influence on its migration into water (distilled or acidic) simulants. This result is coherent with that observed in a previous study [35] on the release kinetics of the compounds in aqueous food simulants at 20 °C, in which the equilibrium release value increased as the compound concentration in the film rose. This indicates that the partition coefficient of the active compound was affected by the different changes produced in the polymer matrix by the differing compound concentration. The higher migration values of active phenolic compounds in acidic water suggest a partial degradation of the polymer matrix by polymer hydrolyses, which promoted compound delivery.

## 4. Conclusions

The incorporation of ferulic and p-coumaric acids into PHBV matrices by melt blending gave rise to different structural arrangements of the compounds, mainly due to their different melting points. While ferulic acid was homogenously mixed in the liquid state with the polymer, p-coumaric acid was present as dispersed particles that did not melt during blending at 170 °C. Both compounds enhanced the barrier capacity of the PHBV films, mainly for oxygen with 9% PCA. Films with FA became more extensible, with a small reduction in the elastic modulus and resistance to break, whereas those containing 9% PCA were more brittle and stiffer. Films with PCA gained opacity as the concentration of dispersed PCA particles increased, whereas FA enhanced the film transparency. The changes promoted in the physical properties of the films were explained by different compound interactions with the polymer chains, the structural arrangement and the modification of polymer crystallinity, which was more noticeable for the homogeneously mixed FA. This compound improved the thermal stability of the polymer at 3 and 6%.

The overall migration of every film formulation was lower than the OML, whereas the release of active compounds was dependent on the food simulant; full release was noted in simulants containing ethanol but was more limited in aqueous systems. Therefore, these films could be used as food contact materials, helping to extend food shelf lives. It is necessary to validate these materials in real foods in order to evaluate their potential as active food packaging for the purposes of preventing oxidative and microbial spoilage and preserving food quality and safety.

## Figures and Tables

**Table 1:** Melting temperature (T_m_) and enthalpy (∆H_m_) of the different blend films obtained in the first and second heating steps of DSC analyses. The glass transition temperature (T_g_) and degree of crystallinity (χ_c_) were obtained from the first heating step of DSC.

	First Heating				Second Heating	
Film	T_g_ (°C)	T_m1_ (°C)	∆H_m1_ (J/g PHBV)	ꭓ_c_ (%)	T_m2_ (°C)	∆H_m2_ (J/g PHBV)
**PHBV**	7.2 ± 0.0 ^c^	171.3 ± 1.0 ^a^	85.1 ± 3.0 ^c^	64.4 ± 0.2 ^a^	170.2 ± 0.4 ^a^	88.0 ± 6.0 ^bc^
**3FA**	7.5 ± 0.2 ^c^	167.8 ± 0.3 ^b^	76.0 ± 2.0 ^a^	57.8 ± 1.5 ^c^	166.3 ± 0.7 ^b^	71.1 ± 1.2 ^a^
**6FA**	9.0 ± 0.2 ^b^	163.6 ± 0.9 ^c^	75.9 ± 0.2 ^ab^	57.5 ± 0.1 ^bc^	161.6 ± 0.5 ^c^	69.7 ± 0.3 ^a^
**9FA**	15.0 ± 0.2 ^a^	163.7 ± 4.9 ^c^	75.5 ± 0.5 ^a^	57.2 ± 0.4 ^c^	157.8 ± 0.0 ^e^	70.7 ± 0.1 ^a^
**3PCA**	7.1 ± 0.7 ^c^	167.9 ± 0.4 ^b^	88.0 ± 9.0 ^c^	66.5 ± 7.5 ^a^	166.2 ± 1.2 ^b^	92.0 ± 6.0 ^c^
**6PCA**	5.3 ± 0.5 ^d^	163.1 ± 0.3 ^c^	83.7 ± 0.4 ^bc^	63.4 ± 0.4 ^ab^	159.8 ± 0.7 ^d^	87.4 ± 0.0 ^bc^
**9PCA**	7.0 ± 0.0 ^c^	158.8 ± 0.7 ^d^	84.7 ± 1.3 ^c^	64.1 ± 1.0 ^a^	154.4 ± 0.7 ^f^	82.6 ± 0.1 ^b^

Superscript letters indicate significant differences between the formulations (*p* < 0.05).

**Table 2:** Mean values and standard deviation of the film thickness (t), water vapour permeability (WVP), oxygen permeability (OP), elastic modulus (EM), tensile strength (TS), elongation at break (E%) and colour coordinates: lightness (L*), hue (h_ab_*) and chroma (C_ab_*).

	PHBV	3FA	6FA	9FA	3PCA	6PCA	9PCA
t (µm)	160 ± 16 ^a^	152 ± 24 ^ab^	152 ± 16 ^bc^	137 ± 15 ^de^	144 ± 12 ^bcd^	143 ± 23 ^cd^	131 ± 12 ^e^
WVP × 10^12^ (g/msPa)	5.4 ± 0.4 ^a^	4.2 ± 0.7 ^bcd^	4.5 ± 0.4 ^bc^	3.4 ± 0.1 ^e^	3.8 ± 0.3 ^cde^	4.7 ± 0.2 ^ab^	3.6 ± 0.3 ^de^
OP × 10^13^ (cm^3^/msPa)	3.70 ± 0.20 ^a^	3.00 ± 0.20 ^b^	3.00 ± 0.04 ^b^	2.78 ± 0.14 ^b^	2.39 ± 0.03 ^c^	2.70 ± 0.20 ^b^	1.26 ± 0.06 ^d^
EM (MPa)	1520 ± 90 ^a^	1480 ± 50 ^a^	1280 ± 40 ^b^	1060 ± 90 ^c^	1480 ± 70 ^a^	1260 ± 70 ^b^	1490 ± 50 ^a^
TS (MPa)	37 ± 2 ^a^	37 ± 2 ^a^	33 ± 3 ^b^	31 ± 4 ^b^	38 ± 2 ^a^	38 ± 2 ^a^	28 ± 7 ^c^
E%	3.6 ± 0.4 ^c^	4.6 ± 0.7 ^b^	4.7 ± 0.8 ^b^	4.2 ± 0.7 ^b^	4.2 ± 0.5 ^b^	5.6 ± 0.7 ^a^	2.4 ± 1.0 ^d^
L*	83.9 ± 0.4 ^b^	81.7 ± 0.5 ^d^	80.8 ± 0.3 ^e^	81.0 ± 0.5 ^e^	82.0 ± 0.2 ^cd^	82.1 ± 0.4 ^b^	85.1 ± 0.5 ^a^
C_ab_*	13.5 ± 0.3 ^e^	14.0 ± 0.4 ^d^	14.7 ± 0.5 ^c^	15.9 ± 0.5 ^ab^	15.5 ± 0.4 ^b^	16.0 ± 0.7 ^a^	11.8 ± 0.5 ^f^
h_ab_*	89.8 ± 0.5 ^a^	87.9 ± 0.4 ^c^	86.6 ± 0.5 ^e^	86.3 ± 0.7 ^e^	89.7 ± 0.4 ^ab^	89.3 ± 0.4 ^b^	87.4 ± 0.8 ^d^

Superscript letters in each column indicate significant differences between film samples (*p* < 0.05).

## Data Availability

Data is contained within the article and also available on request.

## References

[1] Ryberg M.W., Hauschild M.Z., Wang F., Averous-Monnery S., Laurent A. (2019). Global Environmental Losses of Plastics across Their Value Chains. Resour. Conserv. Recycl..

[2] Phelan A., Meissner K., Humphrey J., Ross H. (2022). Plastic Pollution and Packaging: Corporate Commitments and Actions from the Food and Beverage Sector. J. Clean. Prod..

[3] Shlush E., Davidovich-Pinhas M. (2022). Bioplastics for Food Packaging. Trends Food Sci. Technol..

[4] Rillig M.C., Kim S.W., Kim T.-Y., Waldman W.R. (2021). The Global Plastic Toxicity Debt. Environ. Sci. Technol..

[5] Kaur L., Khajuria R., Parihar L., Dimpal Singh G. (2017). Polyhydroxyalkanoates: Biosynthesis to commercial production—A review. J. Microbiol. Biotechnol. Food Sci..

[6] Corre Y.M., Bruzaud S., Audic J.L., Grohens Y. (2012). Morphology and Functional Properties of Commercial Polyhydroxyalkanoates: A Comprehensive and Comparative Study. Polym. Test..

[7] Laycock B., Halley P., Pratt S., Werker A., Lant P. (2013). The Chemomechanical Properties of Microbial Polyhydroxyalkanoates. Prog. Polym. Sci..

[8] Bonnenfant C., Gontard N., Aouf C. (2022). Biobased and Biodegradable Polymers in a Circular Economy Context: Understanding Quercetin and Gallic Acid Impacts on PHBV Thermal Properties. Polym. Degrad. Stab..

[9] Bonnenfant C., Chatellard L., Gontard N., Aouf C. (2023). Effect of Quercetin and Gallic Acid on the Microbial Degradation of Poly(3-Hydroxybutyrate-Co-3-Hydroxyvalerate) (PHBV) Materials. J. Polym. Environ..

[10] Müller K. Active packaging concepts-are they able to reduce food waste?. Proceedings of the 5th International Workshop Cold Chain Management.

[11] Latos-Brozio M., Masek A. (2020). The Application of (+)-Catechin and Polydatin as Functional Additives for Biodegradable Polyesters. Int. J. Mol. Sci..

[12] Auriemma M., Piscitelli A., Pasquino R., Cerruti P., Malinconico M., Grizzuti N. (2015). Blending Poly(3-Hydroxybutyrate) with Tannic Acid: Influence of a Polyphenolic Natural Additive on the Rheological and Thermal Behavior. Eur. Polym. J..

[13] Ordoñez R., Atarés L., Chiralt A. (2022). Biodegradable Active Materials Containing Phenolic Acids for Food Packaging Applications. Compr. Rev. Food Sci. Food Saf..

[14] Gijsman P. (2012). Polymer Stabilization. Handbook of Environmental Degradation of Materials.

[15] Requena R., Vargas M., Chiralt A. (2017). Release Kinetics of Carvacrol and Eugenol from Poly(Hydroxybutyrate-Co-Hydroxyvalerate) (PHBV) Films for Food Packaging Applications. Eur. Polym. J..

[16] Requena R., Vargas M., Chiralt A. (2019). Eugenol and Carvacrol Migration from PHBV Films and Antibacterial Action in Different Food Matrices. Food Chem..

[17] Arrieta M., Díez García A., López D., Fiori S., Peponi L. (2019). Antioxidant Bilayers Based on PHBV and Plasticized Electrospun PLA-PHB Fibers Encapsulating Catechin. Nanomaterials.

[18] Hernández-García E., Vargas M., Chiralt A. (2022). Starch-Polyester Bilayer Films with Phenolic Acids for Pork Meat Preservation. Food Chem..

[19] Latos-Brozio M., Masek A. (2020). Biodegradable Polyester Materials Containing Gallates. Polymers.

[20] Fei B., Chen C., Wu H., Peng S., Wang X., Dong L. (2004). Comparative Study of PHBV/TBP and PHBV/BPA Blends. Polym. Int..

[21] Fei B., Chen C., Wu H., Peng S., Wang X., Dong L., Xin J.H. (2004). Modified Poly(3-Hydroxybutyrate-Co-3-Hydroxyvalerate) Using Hydrogen Bonding Monomers. Polymer.

[22] Xiang H.X., Chen S.H., Cheng Y.H., Zhou Z., Zhu M.F. (2013). Structural Characteristics and Enhanced Mechanical and Thermal Properties of Full Biodegradable Tea Polyphenol/Poly(3-Hydroxybutyrate-Co-3-Hydroxyvalerate) Composite Films. Express Polym. Lett..

[23] Lima M.C., Paiva de Sousa C., Fernandez-Prada C., Harel J., Dubreuil J.D., de Souza E.L. (2019). A Review of the Current Evidence of Fruit Phenolic Compounds as Potential Antimicrobials against Pathogenic Bacteria. Microb. Pathog..

[24] Pernin A., Bosc V., Maillard M.-N., Dubois-Brissonnet F. (2019). Ferulic Acid and Eugenol Have Different Abilities to Maintain Their Inhibitory Activity Against Listeria Monocytogenes in Emulsified Systems. Front. Microbiol..

[25] Dędek K., Rosicka-Kaczmarek J., Nebesny E., Kowalska G. (2019). Characteristics and Biological Properties of Ferulic Acid. Biotechnol. Food Sci..

[26] Lou Z., Wang H., Rao S., Sun J., Ma C., Li J. (2012). P-Coumaric Acid Kills Bacteria through Dual Damage Mechanisms. Food Control.

[27] Mitani T., Ota K., Inaba N., Kishida K., Koyama H.A. (2018). Antimicrobial Activity of the Phenolic Compounds of Prunus Mume against Enterobacteria. Biol. Pharm. Bull..

[28] Miyague L., Macedo R.E.F., Meca G., Holley R.A., Luciano F.B. (2015). Combination of Phenolic Acids and Essential Oils against Listeria Monocytogenes. LWT—Food Sci. Technol..

[29] Shi C., Zhang X., Sun Y., Yang M., Song K., Zheng Z., Chen Y., Liu X., Jia Z., Dong R. (2016). Antimicrobial Activity of Ferulic Acid against Cronobacter Sakazakii and Possible Mechanism of Action. Foodborne Pathog. Dis..

[30] Takahashi H., Kashimura M., Koiso H., Kuda T., Kimura B. (2013). Use of Ferulic Acid as a Novel Candidate of Growth Inhibiting Agent against Listeria Monocytogenes in Ready-to-Eat Food. Food Control.

[31] Boz H. (2015). P-Coumaric Acid in Cereals: Presence, Antioxidant and Antimicrobial Effects. Int. J. Food Sci. Technol..

[32] Hernández-García E., Vargas M., Chiralt A. (2022). Effect of Active Phenolic Acids on Properties of PLA-PHBV Blend Films. Food Packag. Shelf Life.

[33] Yang F., Li Z., Qiu Z. (2011). Miscibility and Crystallization Behavior of Biodegradable Poly(3-Hydroxybutyrate-Co-3-Hydroxyvalerate)/Phenolic Blends. J. Appl. Polym. Sci..

[34] Otero-Pazos P., Rodríguez-Bernaldo De Quirósquirós A., Sendón R.S., Benito-Peñ E., Gonzaíez-Vallejo V., Cruz Moreno-Bondi M., Angulo I., Paseiro-Losada P. (2014). Active Food Packaging Based on Molecularly Imprinted Polymers: Study of the Release Kinetics of Ferulic Acid. J. Agric. Food Chem..

[35] Moll E., González-Martínez C., Chiralt A. (2023). Release and Antibacterial Action of Phenolic Acids Incorporated into PHBV Films. Food Packag. Shelf Life.

[36] Ordoñez R., Atarés L., Chiralt A. (2021). Physicochemical and Antimicrobial Properties of Cassava Starch Films with Ferulic or Cinnamic Acid. LWT.

[37] Miguel O., Egiburu J.L., Iruin J.J. (2001). Blends of Bacterial Poly(3-Hydroxybutyrate) with Synthetic Poly(3-Hydroxybutyrate) and Poly(Epichlorohydrin): Transport Properties of Carbon Dioxide and Water Vapour. Polymer.

[38] (2002). Standard Test Method for Tensile Properties of Thin Plastic Sheeting.

[39] (2010). Standard Test Method for Oxygen Gas Transmission Rate Through Plastic Film and Sheeting Using a Coulometric Sensor.

[40] (2009). Standard Test Methods for Water Vapor Transmission of Materials.

[41] Cano A., Jiménez A., Cháfer M., Gónzalez C., Chiralt A. (2014). Effect of Amylose:Amylopectin Ratio and Rice Bran Addition on Starch Films Properties. Carbohydr. Polym..

[42] Hutchings J.B. (1999). Food and Colour Appearance, Chapman and Hall Food Science Book.

[43] (2002). Materiales y Artículos en Contacto con Productos Alimenticios. Plásticos. Parte 1: Guía para la Elección de Condiciones y Métodos de Ensayo para la Migración Global.

[44] (2022). Materiales y Artículos en Contacto con Productos Alimenticios. Plásticos. Parte 2: Métodos de Ensayo para la Migración Global en Aceites Vegetales.

[45] (2022). Materiales y Artículos en Contacto con Productos Alimenticios. Plásticos. Parte 3: Métodos de Ensayo para la Migración Global en Simulantes Evaporables.

[46] Ordoñez R., Atarés L., Chiralt A. (2022). Effect of Ferulic and Cinnamic Acids on the Functional and Antimicrobial Properties in Thermo-Processed PLA Films. Food Packag. Shelf Life.

[47] Vilas-Boas S.M., Alves R.S., Brandão P., Campos L.M.A., Coutinho J.A.P., Pinho S.P., Ferreira O. (2020). Solid-Liquid Phase Equilibrium of Trans-Cinnamic Acid, p-Coumaric Acid and Ferulic Acid in Water and Organic Solvents: Experimental and Modelling Studies. Fluid. Phase Equilib..

[48] Kaniuk Ł., Ferraris S., Spriano S., Luxbacher T., Krysiak Z., Berniak K., Zaszczynska A., Marzec M.M., Bernasik A., Sajkiewicz P. (2021). Time-Dependent Effects on Physicochemical and Surface Properties of PHBV Fibers and Films in Relation to Their Interactions with Fibroblasts. Appl. Surf. Sci..

[49] Kim G.-M., Michler G.H., Henning S., Radusch H.-J., Wutzler A. (2007). Thermal and Spectroscopic Characterization of Microbial Poly(3-Hydroxybutyrate) Submicrometer Fibers Prepared by Electrospinning. J. Appl. Polym. Sci..

[50] Singh S., Mohanty A.K., Sugie T., Takai Y., Hamada H. (2008). Renewable Resource Based Biocomposites from Natural Fiber and Polyhydroxybutyrate-Co-Valerate (PHBV) Bioplastic. Compos. Part. A Appl. Sci. Manuf..

[51] Bai J., Dai J., Li G. (2015). Electrospun Composites of PHBV/Pearl Powder for Bone Repairing. Prog. Nat. Sci. Mater. Int..

[52] Gonçalves S.P.C., Martins-Franchetti S.M., Chinaglia D.L. (2009). Biodegradation of the Films of PP, PHBV and Its Blend in Soil. J. Polym. Environ..

[53] Chen Y., Zou C., Mastalerz M., Hu S., Gasaway C., Tao X. (2015). Applications of Micro-Fourier Transform Infrared Spectroscopy (FTIR) in the Geological Sciences—A Review. Int. J. Mol. Sci..

[54] Panda P.K., Yang J.-M., Chang Y.-H., Su W.-W. (2019). Modification of Different Molecular Weights of Chitosan by P-Coumaric Acid: Preparation, Characterization and Effect of Molecular Weight on Its Water Solubility and Antioxidant Property. Int. J. Biol. Macromol..

[55] Ramorobi L.M., Matowane G.R., Mashele S.S., Bonnet S.L., Noreljaleel A.E.M., Swain S.S., Makhafola T.J., Chukwuma C.I. (2022). Bioactive Synergism between Zinc Mineral and P-coumaric Acid: A Multi-mode Glycemic Control and Antioxidative Study. J. Food Biochem..

[56] Wang J., Cao Y., Sun B., Wang C. (2011). Characterisation of Inclusion Complex of Trans-Ferulic Acid and Hydroxypropyl-β-Cyclodextrin. Food Chem..

[57] Wang S., Kong L., Zhao Y., Tan L., Zhang J., Du Z., Zhang H. (2019). Lipophilization and Molecular Encapsulation of P-Coumaric Acid by Amylose Inclusion Complex. Food Hydrocoll..

[58] Woranuch S., Yoksan R. (2013). Preparation, Characterization and Antioxidant Property of Water-Soluble Ferulic Acid Grafted Chitosan. Carbohydr. Polym..

[59] González-Ausejo J., Sanchez-Safont E., Lagaron J.M., Olsson R.T., Gamez-Perez J., Cabedo L. (2017). Assessing the Thermoformability of Poly(3-Hydroxybutyrate-Co-3-Hydroxyvalerate)/Poly(Acid Lactic) Blends Compatibilized with Diisocyanates. Polym. Test..

[60] Chikh A., Benhamida A., Kaci M., Pillin I., Bruzaud S. (2016). Synergistic Effect of Compatibilizer and Sepiolite on the Morphology of Poly(3-Hydroxybutyrate-Co-3-Hydroxyvalerate)/Poly(Butylene Succinate) Blends. Polym. Test..

[61] Feijoo P., Samaniego-Aguilar K., Sánchez-Safont E., Torres-Giner S., Lagaron J.M., Gamez-Perez J., Cabedo L. (2022). Development and Characterization of Fully Renewable and Biodegradable Polyhydroxyalkanoate Blends with Improved Thermoformability. Polymers.

[62] Furushima Y., Schick C., Toda A. (2018). Crystallization, Recrystallization, and Melting of Polymer Crystals on Heating and Cooling Examined with Fast Scanning Calorimetry. Polym. Cryst..

[63] Sato H., Nakamura M., Padermshoke A., Yamaguchi H., Terauchi H., Ekgasit S., Noda I., Ozaki Y. (2004). Thermal Behavior and Molecular Interaction of Poly(3-Hydroxybutyrate-*co*-3-Hydroxyhexanoate) Studied by Wide-Angle X-Ray Diffraction. Macromolecules.

[64] Liu W.J., Yang H.L., Wang Z., Dong L.S., Liu J.J. (2002). Effect of Nucleating Agents on the Crystallization of Poly(3-Hydroxybutyrate-Co-3-Hydroxyvalerate). J. Appl. Polym. Sci..

[65] Buzarovska A., Bogoeva-Gaceva G., Grozdanov A.A., Avella A.M., Gentile A.G., Errico A.M. (2007). Crystallization Behavior of Poly(Hydroxybytyrate-Co-Valerate) in Model and Bulk PHBV/Kenaf Fiber Composites. J. Mater. Sci..

[66] Eraslan K., Aversa C., Nofar M., Barletta M., Gisario A., Salehiyan R., Goksu Y.A. (2022). Poly(3-Hydroxybutyrate-Co-3-Hydroxyhexanoate) (PHBH): Synthesis, Properties, and Applications—A Review. Eur. Polym. J..

[67] Naphade R., Jog J. (2012). Electrospinning of PHBV/ZnO Membranes: Structure and Properties. Fibers Polym..

[68] Shuai C., Wang C., Qi F., Peng S., Yang W., He C., Wang G., Qian G. (2020). Enhanced Crystallinity and Antibacterial of PHBV Scaffolds Incorporated with Zinc Oxide. Hindawi J. Nanomater..

[69] Nanda M.R., Misra M., Mohanty A.K. (2011). The Effects of Process Engineering on the Performance of PLA and PHBV Blends. Macromol. Mater. Eng..

[70] Dey A., Neogi S. (2019). Oxygen Scavengers for Food Packaging Applications: A Review. Trends Food Sci. Technol..

[71] Requena R., Jiménez A., Vargas M., Chiralt A. (2016). Effect of Plasticizers on Thermal and Physical Properties of Compression-Moulded Poly[(3-Hydroxybutyrate)-Co-(3-Hydroxyvalerate)] Films. Polym. Test..

[72] Official Journal of the European Union and European Food Safety Authority, “Commission Regulation (EC) No 450/2009 of 29 May 2009 on Active and Intelligent Materials and Articles Intended to Come into Contact with Food,” May 2009, Brussels, Belgium. https://eur-lex.europa.eu/LexUriServ/LexUriServ.do?uri=OJ:L:2009:135:0003:0011:EN:PDF.

[73] Official Journal of the European Union and European Food Safety Authority; “Commission Regulation (EC) No 1935/2004 of 27 October 2004 on Materials and Articles Intended to Come into Contact with Food”, October 2004, Brussels, Belgium. https://eur-lex.europa.eu/legal-content/EN/TXT/PDF/?uri=CELEX:32004R1935.

[74] Angellier-Coussy H., Kemmer D., Gontard N., Peyron S. (2020). Physical–Chemical and Structural Stability of PHBV/Wheat Straw Fibers Based Biocomposites under Food Contact Conditions. J. Appl. Polym. Sci..

[75] Chea V., Angellier-Coussy H., Peyron S., Kemmer D., Gontard N. (2016). Poly(3-Hydroxybutyrate-Co-3-Hydroxyvalerate) Films for Food Packaging: Physical-Chemical and Structural Stability under Food Contact Conditions. J. Appl. Polym. Sci..

[76] Lajarrige A., Gontard N., Gaucel S., Peyron S. (2020). Evaluation of the Food Contact Suitability of Aged Bio-Nanocomposite Materials Dedicated to Food Packaging Applications. Appl. Sci..

[77] Yang W., Fortunati E., Dominici F., Giovanale G., Mazzaglia A., Balestra G.M., Kenny J.M., Puglia D. (2016). Effect of Cellulose and Lignin on Disintegration, Antimicrobial and Antioxidant Properties of PLA Active Films. Int. J. Biol. Macromol..

[78] Sandoval G., Quintana P.G., Baldessari A., Ballesteros A.O., Plou F.J. (2015). Lipase-Catalyzed Preparation of Mono- and Diesters of Ferulic Acid. Biocatal. Biotransform..

[79] Shakeel F., Salem-Bekhit M.M., Haq N., Siddiqui N.A. (2017). Solubility and Thermodynamics of Ferulic Acid in Different Neat Solvents: Measurement, Correlation and Molecular Interactions. J. Mol. Liq..

[80] Ji W., Meng Q., Li P., Yang B., Wang F., Ding L., Wang B. (2016). Measurement and Correlation of the Solubility of P-Coumaric Acid in Nine Pure and Water + Ethanol Mixed Solvents at Temperatures from 293.15 to 333.15 K. J. Chem. Eng. Data.

[81] Jamshidian M., Tehrany E.A., Desobry S. (2012). Release of Synthetic Phenolic Antioxidants from Extruded Poly Lactic Acid (PLA) Film. Food Control.

[82] Iñiguez-Franco F., Auras R., Burgess G., Holmes D., Fang X., Rubino M., Soto-Valdez H. (2016). Concurrent Solvent Induced Crystallization and Hydrolytic Degradation of PLA by Water-Ethanol Solutions. Polymer.

